# A combination of Beers and STOPP criteria better detects potentially inappropriate medications use among older hospitalized patients with chronic diseases and polypharmacy: a multicenter cross-sectional study

**DOI:** 10.1186/s12877-023-03743-2

**Published:** 2023-01-25

**Authors:** Jing Tang, Ke Wang, Kun Yang, Dechun Jiang, Xianghua Fang, Su Su, Yang Lin, Shicai Chen, Hongyan Gu, Pengmei Li, Suying Yan

**Affiliations:** 1 Department of Pharmacy, Xuanwu Hospital, the First Clinical Medical College of Capital Medical University, No. 45, Changchun Street, Xicheng District 100053 Beijing, China; 2 National Clinical Research Center for Geriatric Disorders, Beijing, 100053 China; 3 Department of Evidence-Based Medicine, Xuanwu Hospital, the First Clinical Medical College of Capital Medical University, Beijing, 100053 China; 4 Department of Pharmacy, Beijing Anzhen Hospital Affiliated to Capital Medical University, Beijing, 100029 China; 5 Department of Pharmacy, Beijing Luhe Hospital Affiliated to Capital Medical University, Beijing, 101149 China; 6 Department of Pharmacy, Beijing Shijitan Hospital Affiliated to Capital Medical University, Beijing, 100038 China; 7 Department of Pharmacy, China-Japan Friendship Hospital, Beijing, 100029 China

**Keywords:** Polypharmacy, Inappropriate prescribing, Potentially inappropriate medication, Medication related problems, Aged, Chronic disease

## Abstract

**Background:**

Research on potentially inappropriate medications (PIM) and medication-related problems (MRP) among the Chinese population with chronic diseases and polypharmacy is insufficient.

**Objectives:**

This study aimed to investigate the prevalence of PIM and MRP among older Chinese hospitalized patients with chronic diseases and polypharmacy and analyze the associated factors.

**Methods:**

A retrospective cross-sectional study was conducted in five tertiary hospitals in Beijing. Patients aged ≥ 65 years with at least one chronic disease and taking at least five or more medications were included. Data were extracted from the hospitals’ electronic medical record systems. PIM was evaluated according to the 2015 Beers criteria and the 2014 Screening Tool of Older Persons’ Prescriptions (STOPP) criteria. MRPs were assessed and classified according to the Helper-Strand classification system. The prevalence of PIM and MRP and related factors were analyzed.

**Results:**

A total of 852 cases were included. The prevalence of PIM was 85.3% and 59.7% based on the Beers criteria and the STOPP criteria. A total of 456 MRPs occurred in 247 patients. The most prevalent MRP categories were dosages that were too low and unnecessary medication therapies. Hyperpolypharmacy (taking ≥ 10 drugs) (odds ratio OR 3.736, 95% confidence interval CI 1.541–9.058, *P* = 0.004) and suffering from coronary heart disease (OR 2.620, 95%CI 1.090–6.297, *P* = 0.031) were the influencing factors of inappropriate prescribing (the presence of either PIM or MRP in a patient).

**Conclusion:**

PIM and MRP were prevalent in older patients with chronic disease and polypharmacy in Chinese hospitals. More interventions are urgently needed to reduce PIM use and improve the quality of drug therapies.

**Supplementary Information:**

The online version contains supplementary material available at 10.1186/s12877-023-03743-2.

## Background

The aging of the population presents formidable challenges in healthcare systems, which concerns many countries [1, 2]. Chronic diseases, such as hypertension, dyslipidemia, diabetes, coronary heart disease, and stroke, are common in adults over 65 years of age. About three in four older adults in developed countries live with more than one chronic disease [3]. A similar situation was found in China [4]. For treating these coexisting diseases, older patients often take multiple medications, which leads to polypharmacy. More than half of the older population is exposed to polypharmacy in some settings [5, 6]. Polypharmacy increases the risk of adverse drug reactions, drug-drug interactions, medication non-adherence, and inappropriate use of medications [7–9]. It promotes the emergence of medication-related problems (MRP) [10]. Potentially inappropriate medications (PIMs) are medications whose adverse risk exceed their health benefits when prescribed in older adults [11]. They are associated with adverse clinical outcomes such as falls and risk of frailty in the elderly and can lead to higher utilization of healthcare resources and hospital admissions [12]. Furthermore, patients with MRPs have an increased incidence of drug-related adverse events that lead to a higher risk of mortality [13].

There are multiple screening tools to help healthcare providers select medication therapy and reduce the exposure of older adults to PIM use (PIMU). Among them, the American Geriatric Society (AGS) Beers Criteria [14] and Screening Tool of Older Persons’ Prescriptions (STOPP) are the two most widely used criteria [15]. The Beers criteria was initially published in 1991 by the American Geriatrics Society and the latest version has been available since 2019 [16]. The STOPP criteria was first launched by geriatricians from Cork University Hospital (Ireland) in 2008 and was updated in 2014 [15]. Most published studies on PIMU and MRPs focused on investigating the incidences and factors influencing them. Only a few studies explored the relationship between chronic diseases and PIMU. An investigation determined and assessed the magnitude and predictors of PIMU in older adult patients at the chronic care clinic in southwest Ethiopia [17]. According to Beers and STOPP criteria, at least one PIMU was identified in 83.1% and 45.2% of the patients, respectively. The risk of PIMU according to the Beers criteria increased with age, hypertension, and polypharmacy. Using STOPP criteria, hypertension, diabetes, ischemic heart disease, peripheral neuropathy, and polypharmacy significantly increased the risk of PIMU. Another study conducted in the United States investigated the associations between chronic illness, polypharmacy, and MRPs among Medicare beneficiaries (65 years and older) [18]. The study found that beneficiaries with certain conditions were more likely to suffer from MRP than those without, including depression, congestive heart failure, diabetes, end-stage renal disease, respiratory disorders, and hypertension. Medicare beneficiaries with 11 or more medications were 1.86 times more likely to experience an MRP than those taking fewer medications.

Several studies have investigated PIMU and MRP in Chinese older adults [19–22]. These studies have revealed a high prevalence of PIMU and MRP in aging adult populations. However, information on PIMU and MRP and related factors in older adults with chronic diseases and polypharmacy are still limited. Here, we conducted a multicenter cross-sectional study to investigate the prevalence of PIMU and MRP among older Chinese hospitalized patients with chronic diseases and polypharmacy. We also sought to identify factors associated with PIMU and MRP among the study population to help clinicians manage at-risk patients by improving the rational use of medications.

## Methods

### Study design and population

This retrospective study was conducted in five tertiary hospitals in Beijing (Xuanwu Hospital, Shijitan Hospital, Anzhen Hospital, China-Japan Friendship Hospital, and Luhe Hospital). We included patients discharged from the Department of Neurology, Geriatrics, Cardiology, and Endocrinology in the first week of each month in March, June, September, and December 2017. The study inclusion criteria were 1) patients 65 years or older, and 2) had at least one of the following five chronic diseases as discharge diagnoses: hypertension, hyperlipidemia, coronary heart disease, cerebral infarction, and type 2 diabetes. Only polypharmacy or hyperpolypharmacy patients were included in the analysis. Polypharmacy was defined as the concurrent use of five to nine drugs during hospitalization, and hyperpolypharmacy was defined as taking 10 or more drugs [23]. Patients were excluded from the study if they were hospitalized for less than 48 h, admitted multiple times within a month, or transferred from or to the intensive care unit.

### Data collection

The study data were obtained from the hospitals’ electronic medical record systems. We designed the case report form and used the Research Electronic Data Capture (REDCap) system to manage the form electronically. Data quality control was conducted regularly. The following data were collected: the patient’s personal information (date of birth, gender, height, and weight), hospitalization information (discharge department, date of admission, date of discharge, past medical history, discharge diagnoses, type of health insurance, and laboratory test results) and medication information (drug name, indications, dosage, and adverse reactions). Information concerning traditional Chinese medicines, solvents, temporary medication orders, topical drugs, and hospital preparations was not collected as PIM criteria usually do not apply to these. The Charlson Comorbidity Index (CCI) score was calculated based on the diagnoses at discharge [24]. Drugs were classified according to the World Health Organization Anatomical Therapeutic Chemical (ATC) Classification System (http://www.whocc.no/atc_ddd_index). Each patient was assigned a unique code. All data were de-identified.

### The assessment of PIMU and MRP

PIMU was assessed separately according to the 2015 AGS Beers Criteria and the 2014 STOPP Criteria, the latest versions available at the time of research. Taking at least one item (drug or drug class) listed in either criterion was considered PIMU. The following Beers criteria items (total 87) were assessed: category A-avoided by most older people, category B-avoided by older people with specific health conditions (drug-disease or drug-syndrome interactions), category C-medications to be used with caution, category D-avoided in combination with other treatments because of the risk of harmful “drug-drug” interactions, and category E-dosed differently or avoided among people with reduced kidney function (Supplemental Table 1). Not all STOPP items could be appropriately assessed based on available clinical information. Therefore, the expert panel of the study selected 53.1% (43/81) of the STOPP criteria items for PIM identification (Supplemental Table 2).

**Table 1:** General characteristics of the studied population *n* = 852

Characteristics	Value
Age (year)	74 (69, 80)
Gender	
Female	431(50.6%)
Male	421(49.4%)
Department	
Geriatrics	84 (9.9%)
Endocrinology	97 (11.4%)
Neurology	269 (31.6%)
Cardiology	402 (47.2%)
Medical insurance type	
With health insurance	768 (90.1%)
No health insurance	84 (9.9%)
Length of hospital stays (day)	9(7,13)
Number of diagnoses at discharge	8(6,11)
Number of drugs	10(7, 12)
Hyperpolypharmacy (taking ≥ 10 drugs)	437(51.3%)
CCI (point)	2(1,3)
Kind of chronic diseases	9(7,13)
Hypertension	660(77.5%)
Hyperlipidemia	556(65.3%)
Coronary heart disease	505(59.3%)
Type 2 diabetes	401(47.1%)
Cerebral infarction	354(41.5%)
Number of coexisted chronic diseases ^*^	
1	84(9.9%)
2	215(25.2%)
3	300(35.2%)
4	203(23.8%)
5	50(5.9%)

Categorical variables were expressed as n (percentage). Continuous variables did not follow a normal distribution and were expressed as M (P25, P75)

*CCI* Charlson comorbidity index

^*^Chronic diseases refer to five diseases (hypertension, diabetes, coronary heart disease, cerebral infarction, and hyperlipidemia) in this study

**Table 2:** Most prescribed drug classes (TOP 10)

Most prescribed drug classes (ATC)	n	%
Anti-thrombotic agents (B01A)	720	84.5
Lipid modifying agents, plain (C10A)	717	84.2
Other cardiac preparations (C01E)	447	52.5
Beta blocking agents (C07A)	382	44.8
Selective calcium channel blockers with mainly vascular effects (C08C)	344	40.4
Blood glucose lowering drugs, excl. Insulins (A10B)	316	37.1
Drug for peptic ulcer and gastro-oesophageal reflux disease (GORD) (A02B)	296	34.7
Vasodilators used in cardiac diseases (C01D)	289	33.9
Vitamin B12 and folic acid (B03B)	238	27.9
Angiotensin II receptor blockers (ARBs), Plain (C09C)	220	25.8

*ATC* Anatomical Therapeutic Chemical classification system

The MRP was assessed using the Helper-Strand classification. MRPs were classified into six categories (unnecessary medication therapy, need for additional medication therapy, ineffective medication, dosage too low, adverse drug event, and dosage too high) and 26 causes [25]. Medication adherence was not evaluated due to the lack of documentation in electronic medical records. In this study, inappropriate prescribing was the presence of either a PIM or MRP in a patient.

### Statistical analysis

Variables included age, gender, department, number of diagnoses at discharge, number of drugs, health insurance, CCI, length of hospital stay, and chronic conditions. These variables were used to analyze the influencing factors of PIMU and MRP.

Descriptive statistics were used to describe the general characteristics of the study population and the prevalence of PIM and MRP. Categorical variables are expressed as counts and proportions (%). Continuous variables are expressed as medians and 25 and 75 percentiles (P25, P75) because the data did not conform to the normal distribution. The Wilcoxon signed-rank test and the Chi-squared test were used to compare the groups. A logistic regression model was used to analyze the influencing factors of PIMU and MRP. Odds ratios (OR) and 95% confidence intervals (95% CI) were derived from this model. A two-sided *P* value < 0.05 was considered statistically significant. All statistical analyses were performed using SPSS version 21.0 (SPSS, Inc., Chicago, IL).

## Results

### Characteristics of the study population

A total of 852 cases were included in the analysis. Table 1 describes the characteristics of the study population. The median age was 74 years, and 50.6% (431/852) were females. The median CCI score was 2 points. The majority (90.1%, 768/852) had health insurance. The median hospital stay was nine days, and the median number of discharge diagnoses was eight. Hypertension (77.5%, 660/852), hyperlipidemia (65.3%, 556/852), and coronary heart disease (59.3%, 505/852) were the top three chronic diseases. The patients had an average of 2.9 coexisting chronic diseases. The median number of drugs prescribed was 10. A total of 48.7% (415/852) of patients were polypharmacy patients and 51.3% (437/852) were hyperpolypharmacy patients.

Table 2 shows the top ten most prescribed drug classes. More than half of the patients took antithrombotic agents (84.5%, 720/852), lipid-modifying agents (84.2%, 717/852), and other cardiac preparations (52.5%, 447/852).

### The prevalence of potentially inappropriate medication use

The prevalence of PIMU in this study was 93.8% (799/852), an average of 2.7 PIMs per patient. A total of 20.4% (174/852) of patients took one PIM, and 73.4% (625/852) took two or more PIMs (Table 3). According to Beers Criteria, 85.3% (727/852) of the patients received PIMs that included 47.1% (41/87) of the PIM items. Details are shown in Supplemental Table 1. However, according to the STOPP criteria, 59.7% (509/852) of patients received PIMs which included 81.4% (35/43) of the PIM items. Details are shown in Supplemental Table 2.

**Table 3:** Number of inappropriate medication use

Number of PIM	n1 (%)	Number of MRP	n2 (%)
0	53 (6.2)	0	605 (71.0)
1	174 (20.4)	1	134 (15.7)
2	216 (25.4)	2	58 (6.8)
3	184 (21.6)	3	32 (3.8)
4	107 (12.6)	4	12 (1.4)
5	46 (5.4)	5	6 (0.7)
6	29 (3.4)	6	4 (0.5)
7	25 (2.9)	7	0 (0)
8	9 (1.1)	8	1 (0.1)
9	3 (0.4)		
≥ 10	6 (0.7)		

*PIM* potential inappropriate medication, *MRP* medication related problem, PIM was identified from either Beers or STOPP criteria

The PIM prevalence rates were higher in the following Beers criteria items: 1) vasodilators-use with caution, 65.5% (558/852), 2) proton-pump inhibitors (PPI)-avoid scheduled use for > eight weeks, 32.4% (276/852), 3) antipsychotics and diuretics-may exacerbate or cause syndromes of inappropriate antidiuretic hormone secretion or hyponatremia-use with caution, 31.7% (270/852), 4) short-and intermediate-acting benzodiazepines-avoid, 7.6% (65/852), and 5) aspirin for primary prevention of cardiac events-use with caution in adults aged ≥ 80, 4.3% (37/852). Details are shown in Supplemental Table 1.

The following STOPP criteria items had higher prevalence rates: 1) C8 (NSAID with concurrent antiplatelet agent without PPI prophylaxis), 21.7% (185/852), 2) N1 (concomitant use of two or more drugs with antimuscarinic/anticholinergic properties), 10.6% (90/852), 3) A1 (any drug prescribed without an evidence-based clinical indication), 10.3% (88/852), 4) C3 (aspirin plus clopidogrel as secondary stroke prevention), 10.0% (85/852), and 5) K1 (benzodiazepines), 8.2% (70/852). Details are shown in Supplemental Table 2.

### The analysis of medication-related problems

The analysis of the prevalence of MRP is presented in Tables 3 and 4. A total of 247 patients (29%, 247/852) had MRP (144 hyperpolypharmacy patients and 103 polypharmacy patients). A total of 54.3% (134/247) had one MRP. Dosage too low occurred in 41.3% (102/247) of patients followed by 40.9% (101/247) of patients with unnecessary medication therapy. The prevalent rates of unnecessary medication therapy (14.9% *vs.* 8.7%, *P* = 0.005), ineffective medication (3.9% *vs.* 0.7%, *P* = 0.002), dosage too low (14.9% *vs*. 8.9%, *P* = 0.007), and dosage too high (10.1% *vs.* 5.3%, *P* = 0.009) were significantly higher in hyperpolypharmacy patients than in polypharmacy patients.

**Table 4:** Prevalence of medication related problems categories

MRP categories	Total(*N* = 852) N(%)	Number of drugs		P
		**5–9(*N* = 415) N(%)**	** ≥ 10(N = 437) N(%)**	
Unnecessary medication therapy	101(11.9)	36(8.7)	65(14.9)	0.005
Needs additional medication therapy	22(2.6)	11(2.7)	11(2.5)	0.902
Ineffective medication	20(2.3)	3(0.7)	17(3.9)	0.002
Dosage too low	102(12.0)	37(8.9)	65(14.9)	0.007
Adverse drug event	33(3.9)	13(3.1)	20(4.6)	0.275
Dosage too high	66(7.7)	22(5.3)	44(10.1)	0.009

*MRP* medication related problem, N refers to the number of patients with MRP categories

Table 5 shows the analysis of MRP causes. The most common causes of MRPs were no medication indication (28.3%, 129/456), dosage too low to produce the desired response (25.4%, 116/456), and dosage too high (10.1%, 46/456).

**Table 5:** Causes of medication related problems

MRP categories and causes	Total n(%)	Number of drugs	
		**5–9 n(%)**	** ≥ 10 n(%)**
**Unnecessary medication therapy**			
No medical indication	129(28.3)	40(27.0)	89(28.9)
Multiple medications used for a condition that requires single medication	20(4.4)	10(6.8)	10(3.2)
Nondrug therapy more appropriate	3(0.7)	0(0)	3(1.0)
Treating avoidable adverse drug event	3(0.7)	1(0.7)	2(0.6)
**Needs additional medication therapy**			
Medical condition requires the initiation of medication therapy	7(1.5)	4(2.7)	3(1.0)
Preventive medication therapy is required to reduce the risk of developing a new condition	8(1.8)	4(2.7)	4(1.3)
Medical condition requires additional pharmacotherapy to attain synergistic or additive effects	10(2.2)	4(2.7)	6(1.9)
**Ineffective medication**			
Condition refractory to drug	10(2.2)	2(1.4)	8(2.6)
Dosage form inappropriate	6(1.3)	0(0)	6(1.9)
Medication product is not an effective product for the indication being treated	5(1.1)	1(0.7)	4(1.3)
**Dosage too low**			
Dose is too low to produce the desired response	116(25.4)	36(24.3)	80(26.0)
Dosage interval is too infrequent to produce the desired response	10(2.2)	5(3.4)	5(1.6)
Medication interaction reduces the amount of active medication available	0(0)	0(0)	0(0)
Duration of medication therapy is too short to produce the desired response	8(1.8)	1(0.7)	7(2.3)
**Adverse drug event**			
Medication product causes an undesirable reaction that is not dose-related	13(2.9)	4(2.7)	9(2.9)
Safer medication product is required because of risk factors	9(2.0)	3(2.0)	6(1.9)
Drug interaction causes an undesirable reaction that is not dose-related	1(0.2)	1(0.7)	0(0)
Dosage increase or decrease too fast	1(0.2)	1(0.7)	0(0)
Medication product causes an allergic reaction	1(0.2)	0(0)	1(0.3)
Medication product is contraindicated because of risk factors	13(2.9)	5(3.4)	8(2.6)
Inappropriate dosage form is used	5(1.1)	0(0)	5(1.6)
**Dosage too high**			
Dose too high	46(10.1)	14(9.5)	32(10.4)
Dosing interval is too short	29(6.4)	10(6.8)	19(6.2)
Duration of medication therapy is too long	3(0.7)	2(1.4)	1(0.3)
Drug interaction occurs resulting in a toxic reaction to the medication product	0(0)	0(0)	0(0)
Dose of the medication was administered too rapidly	0(0)	0(0)	0(0)
**Total**	**456**	**148**	**308**

*MRP* medication related problem, n refers to the numbers of MRPs

### Factors associated with PIM, MRP, and inappropriate prescribing

Table 6 reports the results of the multivariate logistic regression of PIM, MRP and inappropriate prescribing (the presence of either PIM or MRP). The cardiology department had the highest proportion of patients with PIM and the lowest proportion of patients with MRP and was chosen as the reference department. Inappropriate prescribing was associated with hyperpolypharmacy (OR 3.736, 95%CI 1.541–9.058, *P* = 0.004) and coronary heart disease (OR 2.62, 95%CI 1.09–6.297, *P* = 0.031). Patients attending cardiology had a significantly higher risk of PIM when compared to those attending endocrinology (OR 0.085, 95%CI 0.029–0.247, *P* < 0.01). The occurrence of MRP was most significant in the male gender (OR 1.448, 95%CI 1.057–1.983, *P* = 0.021) and with hyperpolypharmacy (OR 1.583, 95%CI 1.114–2.248, *P* = 0.01). Patients diagnosed with hyperlipidemia had fewer MRPs (OR 0.54, 95%CI 0.386–0.756, *P* < 0.001). Patients attending the geriatrics (OR 3.674, 95%CI 2.089–6.459, *P* < 0.001) and neurology (OR 2.274, 95% CI 1.451–3.566, *P* < 0.001) departments had significantly higher MRP prevalence rates than those attending the cardiology department.

**Table 6:** Multivariable analysis of raleted factors associated with PIM and MRP

Variable	PIM		MRP		Inappropriate Medication Use	
	**OR (95%CI)**	**P**	**OR (95%CI)**	**P**	**OR (95%CI)**	**P**
Age	1.002 (0.955–1.052)	0.935	0.988 (0.964–1.013)	0.344	1.024(0.966–1.086)	0.428
Gender (male)	0.810 (0.432–1.519)	0.511	1.448 (1.057–1.983)	0.021	1.035(0.499–2.150)	0.926
Department						
Cardiology	1.000		1.000		1.000	
Geriatrics	0.361 (0.101–1.283)	0.115	3.674 (2.089–6.459)	< .001	0.668(0.121–3.683)	0.643
Endocrinology	0.085 (0.029–0.247)	< .001	1.323 (0.728–2.405)	0.358	0.068(0.020–0.230)	0.000
Neurology	0.372 (0.132–1.052)	0.062	2.274 (1.451–3.566)	< .001	0.477(0.146–1.566)	0.223
Health insurance	0.745 (0.271–2.046)	0.568	0.881 (0.529–1.467)	0.626	0.577(0.165–2.016)	0.389
Length of hospital stay	1.019 (0.940–1.106)	0.641	0.999 (0.961–1.038)	0.948	1.007(0.917–1.107)	0.878
CCI	1.114 (0.865–1.434)	0.403	0.961 (0.853–1.082)	0.511	1.193(0.868–1.639)	0.276
Number of diagnoses at discharge	0.970 (0.868–1.084)	0.589	1.016 (0.962–1.072)	0.578	1.007(0.881–1.150)	0.924
Number of drugs						
5–9	1.000		1.000		1.000	
≥ 10	2.653 (1.275–5.522)	0.009	1.583 (1.114–2.248)	0.010	3.736(1.541–9.058)	0.004
Chronic conditions						
Hypertension	1.138 (0.568–2.278)	0.716	0.982 (0.672–1.435)	0.925	0.838(0.365–1.922)	0.676
Type 2 diabetes	0.524 (0.237–1.158)	0.110	1.149 (0.800–1.649)	0.452	0.500(0.195–1.283)	0.150
Coronary heart disease	3.002 (1.407–6.408)	0.004	1.141 (0.796–1.638)	0.472	2.620(1.090–6.297)	0.031
Cerebral infarction	1.152 (0.562–2.359)	0.699	1.023 (0.713–1.468)	0.901	1.051(0.442–2.496)	0.991
Hyperlipidemia	1.531 (0.793–2.958)	0.205	0.540 (0.386–0.756)	< .001	1.400(0.651–3.013)	0.389

*PIM* potential inappropriate medication, *MRP* medication related problem, *CCI* Charlson comorbidity index, Inappropriate Medication Use refers to the occurrence of either PIM or MRP; PIM was identified from either Beers or STOPP criteria

## Discussion

This is the first multicenter study analyzing PIM and MRP in Chinese hospitalized patients with chronic disease and polypharmacy or hyperpolypharmacy. The prevalence of PIM was high, 93.8% in this population. The incidence of MRP was 29.0%, and the most prevalent MRP categories were dosage too low and unnecessary medication therapy. Hyperpolypharmacy and the presence of coronary heart disease were the influencing factors of PIMU.

Due to data availability and prescribing habits, PIMU varies significantly between countries, regions, and populations. A study evaluated the incidence of PIMU in older patients hospitalized for chronic disease exacerbation in five hospitals in Spain. The results showed that 81.5% of the patients had at least one PIM [26]. The incidence rate of PIMU was 73.2% according to the 2014 STOPP criteria. Another study analyzed the prevalence of PIMU in older patients in an internal medicine department of a Portuguese hospital. The results showed that according to the 2019 Beers criteria and the 2014 STOPP criteria, 92.0% and 76.5% of the patients used at least one PIM, respectively [27]. An analysis of PIMU for older patients with polypharmacy in nine tertiary hospitals in Chengdu, China, found that the incidence of PIMU was 72.54% according to the 2015 Beers criteria [28]. Compared to these studies, the incidence of PIMU in this study was higher, which could be due to the population being patients with chronic diseases and polypharmacy or hyperpolypharmacy. Since there are overlaps and differences between different PIM judgment criteria [29], the combination of Beers criteria and STOPP criteria can detect more PIMs.

PIMs in this study population differed from our previous study among outpatients [30]. In this study, the most common PIM item according to the Beers criteria was vasodilators. Vasodilators should be used with caution in older adults whose vasodilatory effects causing orthostatic hypotension and exacerbating syncope attacks in patients with a history of syncope. More than half of the study patients had coronary heart disease, and about half were treated with isosorbide mononitrate. Isosorbide mononitrate reduces myocardial oxygen consumption, improves myocardial perfusion, and relieves symptoms of angina pectoris. It is recommended for patients with objective evidence of ischemia [31]. To reduce the risk of hypotension and even falls caused by nitrates, older patients should start with a lower dose, adjust the dose according to treatment response, and pay attention to drug resistance over long-term use [32]. However, vasodilators were removed from the 2019 Beers criteria due to the risk not being unique to older patients [16] and the prevalence of PIM was expected to decrease according to this version criteria. PPIs were the second most commonly prescribed PIM item in this study population, and the incidence is similar to that of other related studies, 11.3%-42.6% [33, 34]. PPIs are commonly used in treating peptic ulcers and chronic gastritis and are also widely used to prevent stress ulcers in hospitalized patients [35]. The long-term use of PPI in older patients leaves the patients more prone to complications such as Clostridium difficile infection or renal toxicity [36]. Therefore, pharmacists should focus on reviewing indications for PPI use and avoid unnecessary use for more than eight weeks. According to the STOPP criteria, the most common PIM item was the combined use of NSAIDs and antiplatelet drugs but without the prophylactic use of PPIs. NSAIDs should be avoided in patients with a high risk of gastrointestinal bleeding. If necessary, cyclooxygenase-2 inhibitors can be selected to be taken with PPIs to prevent drug-related gastrointestinal mucosal injury [37].

The incidence of MRP in this study was 29.0%, lower than that of several other studies which had rates of 63.3% to 70.8% [38, 39]. The rates of MRP occurrences and categories vary based on the study population and the classification tools used. In our study, the analyzed medications were long-term treatments for chronic diseases and might have already been optimized. Furthermore, the MRP evaluation was based on a retrospective review of electronic medical record systems. The most common MRP categories were dosage too low and unnecessary medication therapy. The results suggest that clinicians should not only pay attention to the overtreatment of patients with polypharmacy and streamline prescriptions but also be aware of the potential undertreatment [40, 41]. Suboptimal dosing increases the risk of disease exacerbation leading to increased utilization of healthcare resources [42, 43].

Hypertension, diabetes, coronary heart disease, cerebral infarction, and hyperlipidemia are common chronic diseases in older adults [30]. Comorbidities such as endocrine and metabolic disorders, diabetes, coronary heart disease, and cerebrovascular disease were factors of polypharmacy in older adults [44–46]. Our study investigated PIMU in patients with chronic diseases and polypharmacy or hyperpolypharmacy. The results show that people with coronary heart disease had increased PIMU. Hyperlipidemia might be a protective factor for the development of MRP. However, this was not reflected in the results of the influencing factors of PIMU and needed to be confirmed by subsequent larger sample size studies. Our study found that the occurrence of PIM and MRP was related to the number of drugs. Hyperpolypharmacy was associated with an increase in PIM and MRP. The results are consistent with previous studies [17, 18, 39, 47]. Clinicians should carefully review drug therapies and detect PIMs in patients with hyperpolypharmacy. Unlike other studies [30, 48], the risk of MRP was higher in male patients in our study.

Our study has several limitations. First, the retrospective review may lead to underestimating PIMU and MRP. Second, we only included patients with common chronic diseases, which may cause selection bias. And the setting of inclusion and exclusion criteria may affect the generalizability of the results. Third, we did not use the most recent Beers criteria (2019). Finally, this study included patients from tertiary hospitals in Beijing, and the findings may not represent community hospitals or other areas. More extensive sample and multiregional studies are still needed to support the conclusion of this study.

## Conclusion

The prevalence of PIM and MRP is high in older hospitalized patients with chronic disease and polypharmacy or hyperpolypharmacy. Coronary heart disease and hyperpolypharmacy were associated with an increase in PIMU. More interventions are urgently needed to reduce PIMU in this patient population.

## Supplementary Information

**Additional file 1:** 

**Additional file 2:** 

## Data Availability

The datasets generated and/or analyzed during the current study are not publicly available due to the restriction under the institutional ethical committee’s policy, but may be available from the corresponding author on reasonable request and with permission of the ethical committee.

## Abbreviations

PIM: Potentially inappropriate medications
MRP: Medication-related problems
STOPP: Screening Tool of Older Person’s Prescriptions
PIMU: Potentially inappropriate medication use
AGS: American Geriatric Society
REDCap: Research Electronic Data Capture
CCI: Charlson Comorbidity Index
ATC: Anatomical Therapeutic Chemical
PPI: Proton-pump inhibitors
