# Supplementary material for: A combination of Beers and STOPP criteria better detects potentially inappropriate medications use among older hospitalized patients with chronic diseases and polypharmacy: a multicenter cross-sectional study
Source: BMC Geriatr. 2023 Jan 25;23:44. doi: 10.1186/s12877-023-03743-2 (PMC9875512; doi:10.1186/s12877-023-03743-2)
Supplement: Supplementary file 1 — Additional file 1: [file 12877_2023_3743_MOESM1_ESM.docx]

**Supplementary 1: Beers Criteria items and prevalence rates**

| **Beers Criteria items** | | | | | | | **Prevalence rates (%)** |
| --- | --- | --- | --- | --- | --- | --- | --- |
| **Potentially Inappropriate Medication Use in Older Adults** | | | | | | | |
| **Therapeutic Category, Drugs** | | | | | | **Recommendation** |  |
| **First-generation antihistamines**  Brompheniramine/ Carbinoxamine/ Chlorpheniramine/ Clemastine/ Cyproheptadine/ Dexbrompheniramine/ Dexchlorpheniramine/ Dimenhydrinate/ Diphenhydramine (oral)/ Doxylamine/ Hydroxyzine/ Meclizine/ Promethazine/ Triprolidine. | | | | | | Avoid | 0.4 (3/852) |
| **Antiparkinsonian agents**  Benztropine (oral)/ Trihexyphenidyl | | | | | | Avoid | 0 |
| **Antispasmodics**  Atropine (excludes ophthalmic)/ Belladonna alkaloids/ Clidinium-Chlordiazepoxide/ Dicyclomine/ Hyoscyamine/ Propantheline/ Scopolamine | | | | | | Avoid | 0 |
| **Antithrombotics**  Dipyridamole, oral short-acting (does not apply to the extended- release combination with aspirin) | | | | | | Avoid | 0 |
| Ticlopidine | | | | | | Avoid | 0 |
| **Anti-infective**  Nitrofurantoin | | | | | | Avoid in individuals with creatinine clearance <30 mL/min or for long-term suppression of bacteria | 0 |
| **Peripheral alpha-1 blockers**  Doxazosin/ Prazosin/ Terazosin | | | | | | Avoid use as an antihypertensive | 2.7 (23/852) |
| **Central alpha blockers**  Clonidine/ Guanabenz/ Guanfacine/ Methyldopa/ Reserpine (>0.1 mg/d) | | | | | | Avoid clonidine as first-line antihypertensive Avoid others as listed | 0.1 (1/852) |
| Disopyramide | | | | | | Avoid | 0 |
| Dronedarone | | | | | | Avoid in individuals with permanent atrial fibrillation or severe or recently decompensated heart failure | 0 |
| Digoxin | | | | | | Avoid as first-line therapy for atrial fibrillation and heart failure；If used for atrial fibrillation or heart failure, avoid dosages >0.125 mg/d | 2.9 (25/852) |
| Nifedipine, immediate release | | | | | | Avoid | 0 |
| Amiodarone | | | | | | Avoid amiodarone as first-line therapy for atrial fibrillation unless patient has heart failure or substantial left ventricular hypertrophy | 2.7 (23/852) |
| **Antidepressants, alone or in combination**  Amitriptyline/ Amoxapine/ Clomipramine/ Desipramine/ Doxepin >6 mg/d/ Imipramine/ Nortriptyline/ Paroxetine/ Protriptyline/ Trimipramine | | | | | | Avoid | 0.2 (2/852) |
| Antipsychotics, first- (conventional) and second- (atypical) generation | | | | | | Avoid, except for schizophrenia, bipolar disorder, or short-term use as antiemetic during chemotherapy | 2.2 (19/852) |
| **Barbiturates**  Amobarbital/ Butabarbital/ Butalbital/ Mephobarbital/ Pentobarbital/ Phenobarbital/ Secobarbital | | | | | | Avoid | 0 |
| **Benzodiazepines Short- and intermediate- acting** Alprazolam/ Estazolam/ Lorazepam/ Oxazepam/ Temazepam/ Triazolam | | | | | | Avoid | 7.6 (65/852) |
| **Benzodiazepines Long-acting**  Clorazepate/ Chlordiazepoxide (alone or in combination with amitriptyline or clidinium)/ Clonazepam/ Diazepam/ Flurazepam/ Quazepam | | | | | | Avoid | 0.4 (3/852) |
| Meprobamate | | | | | | Avoid | 0 |
| **Nonbenzodiazepine, benzodiazepine receptor agonist hypnotics**  Eszopiclone/ Zolpidem/ Zaleplon | | | | | | Avoid | 1.1 (9/852) |
| Ergoloid mesylates (dehydrogenated ergot alkaloids) Isoxsuprine | | | | | | Avoid | 0.2 (2/852) |
| **Androgens**  Methyltestosterone/ Testosterone | | | | | | Avoid unless indicated for confirmed hypogonadism with clinical symptoms | 0 |
| Desiccated thyroid | | | | | | Avoid | 0 |
| Estrogens with or without progestins | | | | | | Avoid oral and topical patch Vaginal cream or tablets: acceptable to use low-dose intravaginal estrogen for management of dyspareunia, lower urinary tract infections, and other vaginal symptoms | 0 |
| Growth hormone | | | | | | Avoid, except as hormone replacement after pituitary gland removal | 0 |
| Insulin, sliding scale | | | | | | Avoid | 0 |
| Megestrol | | | | | | Avoid | 0 |
| **Sulfonylureas, long-duration**  Chlorpropamide/ Glyburide | | | | | | Avoid | 0.1 (1/852) |
| Metoclopramide | | | | | | Avoid, unless for gastroparesis | 0 |
| Mineral oil, given orally | | | | | | Avoid | 0 |
| Proton-pump inhibitors | | | | | | Avoid scheduled use for >8 weeks unless for high-risk patients (e.g., oral corticosteroids or chronic NSAID use), erosive esophagitis, Barrett’s esophagitis, pathological hypersecretory condition, or demonstrated need for maintenance treatment (e.g., due to failure of drug discontinuation trial or H2 blockers) | 32.4 (276/852) |
| Meperidine | | | | | | Avoid, especially in individuals with chronic kidney disease | 0 |
| **Non-cyclooxygenase-selective NSAIDs, oral:** Aspirin >325 mg/d/ Diclofenac/ Diflunisal/ Etodolac/ Fenoprofen/ Ibuprofen/ Ketoprofen/ Meclofenamate/ Mefenamic acid/ Meloxicam/ Nabumetone/ Naproxen/ Oxaprozin/ Piroxicam/ Sulindac/ Tolmetin | | | | | | Avoid chronic use, unless other alternatives are not effective and patient can take gastroprotective agent (proton- pump inhibitor or misoprostol) | 0.9 (8/852) |
| Indomethacin | | | | | | Avoid | 0 |
| Ketorolac, includes parenteral | | | | | | Avoid | 0 |
| Pentazocine | | | | | | Avoid | 0 |
| **Skeletal muscle relaxants**  Carisoprodol/ Chlorzoxazone/ Cyclobenzaprine/ Metaxalone/ Methocarbamol/ Orphenadrine | | | | | | Avoid | 0 |
| Desmopressin | | | | | | Avoid for treatment of nocturia or nocturnal polyuria | 0 |
| **Potentially Inappropriate Medication Use in Older Adults Due to Drug–Disease or Drug–Syndrome Interactions That May Exacerbate the Disease or Syndrome** | | | | | | | |
| **Disease or Syndrome** | **Drug(s)** | | | | | **Recommendation** |  |
| Heart failure | NSAIDs and COX-2 inhibitors Nondihydropyridine CCBs (diltiazem, verapamil) —avoid only for heart failure with reduced ejection fraction Thiazolidinediones (pioglitazone, rosiglitazone) Cilostazol Dronedarone (severe or recently decompensated heart failure) | | | | | Avoid | 3.8 (32/852) |
| Syncope | AChEIs  Peripheral alpha-1 blockers (Doxazosin Prazosin Terazosin)  Tertiary TCAs  Chlorpromazine  Thioridazine  Olanzapine | | | | | Avoid | 0.1 (1/852) |
| Chronic seizures or epilepsy | Bupropion Chlorpromazine Clozapine Maprotiline Olanzapine Thioridazine Thiothixene Tramadol | | | | | Avoid | 0 |
| Delirium | Anticholinergics Antipsychotics Benzodiazepines Chlorpromazine Corticosteroidsa  H2-receptor antagonists (Cimetidine Famotidine Nizatidine Ranitidine)  Meperidine  Sedative hypnotics | | | | | Avoid | 0.4 (3/852) |
| Dementia or cognitive impairment | Anticholinergics  Benzodiazepines  H2-receptor antagonists Nonbenzodiazepine, benzodiazepine receptor agonist hypnotics(Eszopiclone Zolpidem Zaleplon)  Antipsychotics, chronic and as-needed use | | | | | Avoid | 0.7 (6/852) |
| History of falls or fractures | Anticonvulsants Antipsychotics Benzodiazepines Nonbenzodiazepine, benzodiazepine receptor agonist hypnotics Eszopiclone Zaleplon Zolpidem TCAs SSRIs Opioids | | | | | Avoid unless safer alternatives are not available; avoid anticonvulsants except for seizure and mood disorders  Opioids: avoid, excludes pain management due to recent fractures or joint replacement | 1.1 (9/852) |
| Insomnia | Oral decongestants (Pseudoephedrine/ Phenylephrine)  Stimulants (Amphetamine/ Armodafinil/ Methylphenidate/ Modafinil) Theobromines (Theophylline/ Caffeine) | | | | | Avoid | 0.2 (2/852) |
| Parkinson disease | All antipsychotics (except aripiprazole, quetiapine, clozapine)  Antiemetics (Metoclopramide/Prochlorperazine/Promethazine) | | | | | Avoid | 0.2 (2/852) |
| History of gastric or duodenal ulcers | Aspirin (>325 mg/d)  Non-COX-2 selective NSAIDs | | | | | Avoid unless other alternatives are not effective and patient can take gastroprotective agent (i.e., proton-pump inhibitor or misoprostol) | 0 |
| Chronic kidney disease Stages IV or less (creatinine clearance <30 mL/min) | NSAIDs (non-COX and COX-selective, oral and parenteral) | | | | | Avoid | 0.9 (8/852) |
| Urinary incontinence (all types) in women | Estrogen oral and transdermal (excludes intravaginal estrogen)  Peripheral alpha-1 blockers (Doxazosin/ Prazosin/ Terazosin) | | | | | Avoid in women | 0 |
| Lower urinary tract symptoms, benign prostatic hyperplasia | Strongly anticholinergic drugs, except antimuscarinics for urinary incontinence | | | | | Avoid in men | 0.8 (7/852) |
| **Potentially Inappropriate Medications to Be Used**  **with Caution in Older Adults** | | | | | | | |
| **Drug(s)** | | | | **Recommendation** | | |  |
| Aspirin for primary prevention of cardiac events | | | | Use with caution in adults aged ≥80 | | | 4.3 (37/852) |
| Dabigatran | | | | Use with caution in in adults aged  ≥75 and in patients with CrCl  <30 mL/min | | | 0.9 (8/852) |
| Prasugrel | | | | Use with caution in adults aged  ≥75 | | | 0 |
| Antipsychotics Diuretics Carbamazepine Carboplatin Cyclophosphamide Cisplatin Mirtazapine Oxcarbazepine SNRIs SSRIs TCAs Vincristine | | | | Use with caution | | | 31.7 (270/852) |
| Vasodilators | | | | Use with caution | | | 65.5 (558/852) |
| **Potentially Clinically Important Non-Anti-infective**  **Drug–Drug Interactions That Should Be Avoided in Older Adults** | | | | | | | |
| **Object Drug and**  **Class** | | **Interacting Drug**  **and Class** | | **Recommendation** | | |  |
| ACEIs | | Amiloride or  triamterene | | Avoid routine use;  reserve for  patients with demonstrated  hypokalemia while taking an ACEI | | | 0 |
| Anticholinergic | | Anticholinergic | | Avoid, minimize number of  anticholinergic drugs | | | 0 |
| Antidepressants (i.e.,  TCAs and SSRIs) | | ≥2 other CNS-active  drugs | | Avoid total of ≥3 CNS-active  drugs; minimize number of CNSactive drugs | | | 0.5 (4/852) |
| Antipsychotics | | ≥2 other CNS-active  drugs | | Avoid total of ≥3 CNS-active  drugs; minimize number of CNSactive drugs | | | 0.1 (1/852) |
| Benzodiazepines and nonbenzodiazepine, benzodiazepine receptor agonist hypnotics | | ≥2 other CNS-active  drugs | | Avoid total of ≥3 CNS-active  drugs; minimize number of CNSactive drugs | | | 0.1 (1/852) |
| Corticosteroids, oral or parenteral | | NSAIDs | | Avoid; if not possible, provide  gastrointestinal protection | | | 1.4 (12/852) |
| Lithium | | ACEIs  Loop diuretics | | Avoid, monitor lithium  concentrations | | | 0 |
| Opioid receptor agonist  analgesics | | ≥2 other CNS-active  drugs | | Avoid total of ≥3 CNS-active  drugs; minimize number of CNS  drugs | | | 0 |
| Peripheral Alpha-1  blockers | | Loop diuretics | | Avoid in older women, unless  conditions warrant both drugs | | | 0.8 (7/852) |
| Theophylline | | Cimetidine | | Avoid | | | 0 |
| Warfarin | | Amiodarone | | Avoid when possible; monitor  international normalized ratio  closely | | | 0.5 (4/852) |
| Warfarin | | NSAIDs | | Avoid when possible; if used  together, monitor for bleeding  closely | | | 1.5 (13/852) |
| **Non-Anti-Infective Medications That Should Be**  **Avoided or Have Their Dosage Reduced with Varying Levels of Kidney Function in Older Adults** | | | | | | | |
| **Medication** | | | **Creatinine Clearance,**  **mL/min, at Which**  **Action Required** | | **Recommendation** | |  |
| Amiloride | | | <30 | | Avoid | | 0 |
| Apixaban | | | <25 | | Avoid | | 0 |
| Dabigatran | | | <30 | | Avoid | | 0.1 (1/852) |
| Edoxaban | | | 30-50  <30 or >95 | | Reduce dose  Avoid | | 0 |
| Enoxaparin | | | <30 | | Reduce dose | | 0 |
| Fondaparinux | | | <30 | | Avoid | | 0 |
| Rivaroxaban | | | 30-50  <30 | | Reduce dose  Avoid | | 0.5 (4/852) |
| Spironolactone | | | <30 | | Avoid | | 1.3 (11/852) |
| Triamterene | | | <30 | | Avoid | | 0 |
| Duloxetine | | | <30 | | Avoid | | 0 |
| Gabapentin | | | <60 | | Reduce dose | | 0.6 (5/852) |
| Levetiracetam | | | ≤80 | | Reduce dose | | 0.4 (3/852) |
| Pregabalin | | | <60 | | Reduce dose | | 0 |
| Tramadol | | | <30 | | Immediate release: reduce  dose  Extended release: avoid | | 0.1 (1/852) |
| Cimetidine | | | <50 | | Reduce dose | | 0 |
| Famotidine | | | <50 | | Reduce dose | | 0.8 (7/852) |
| Nizatidine | | | <50 | | Reduce dose | | 0 |
| Ranitidine | | | <50 | | Reduce dose | | 0 |
| Colchicine | | | <30 | | Reduce dose; monitor for  adverse effects | | 0 |
| Probenecid | | | <30 | | Avoid | | 0 |
