# Supplementary material for: A combination of Beers and STOPP criteria better detects potentially inappropriate medications use among older hospitalized patients with chronic diseases and polypharmacy: a multicenter cross-sectional study
Source: BMC Geriatr. 2023 Jan 25;23:44. doi: 10.1186/s12877-023-03743-2 (PMC9875512; doi:10.1186/s12877-023-03743-2)
Supplement: Supplementary file 2 — Additional file 2: [file 12877_2023_3743_MOESM2_ESM.docx]

**Supplementary 2: STOPP Criteria items used in this study and prevalence rates**

| **STOPP Criteria items** | **Prevalence rates (%)** |
| --- | --- |
| **Section A: Indication of medication** | |
| A1: Any drug prescribed without an evidence-based clinical indication. | 10.3 (88/852) |
| A2: Any drug prescribed beyond the recommended duration, where treatment duration is well defined. | 0.4 (3/852) |
| A3: Any duplicate drug class prescription e.g. two concurrent NSAIDs, SSRIs, loop diuretics, ACE inhibitors, anticoagulants (optimisation of monotherapy within a single drug class should be observed prior to considering a new agent). | 1.9 (16/852) |
| **Section B: Cardiovascular System** | |
| B1: Beta-blocker in combination with verapamil or diltiazem (risk of heart block). | 1.8 (15/852) |
| B2: Beta blocker with bradycardia (< 50/min), type II heart block or complete heart block (risk of complete heart block, asystole). | 0.9 (8/852) |
| B3: Loop diuretic for dependent ankle oedema without clinical, biochemical evidence or radiological evidence of heart failure, liver failure, nephrotic syndrome or renal failure (leg elevation and /or compression hosiery usually more appropriate). | 1.1 (9/852) |
| B4: Thiazide diuretic with current significant hypokalaemia (i.e. serum K+ < 3.0 mmol/l), hyponatraemia (i.e. serum Na+ < 130 mmol/l) hypercalcaemia (i.e. corrected serum calcium > 2.65 mmol/l) or with a history of gout (hypokalaemia, hyponatraemia, hypercalcaemia and gout can be precipitated by thiazide diuretic). | 1.1 (9/852) |
| B5: ACE inhibitors or Angiotensin Receptor Blockers in patients with hyperkalaemia. | 0.5 (4/852) |
| B6: Aldosterone antagonists (e.g. spironolactone, eplerenone) with concurrent potassium-conserving drugs (e.g. ACEI’s, ARB’s, amiloride, triamterene) without monitoring of serum potassium (risk of dangerous hyperkalaemia i.e. > 6.0 mmol/l – serum K should be monitored regularly, i.e. at least every 6 months). | 0.4 (3/852) |
| **Section C: Antiplatelet/Anticoagulant Drugs** | |
| C1: Aspirin with a past history of peptic ulcer disease without concomitant PPI (risk of recurrent peptic ulcer ). | 0.9 (8/852) |
| C2: Aspirin, clopidogrel, dipyridamole, vitamin K antagonists, direct thrombin inhibitors or factor Xa inhibitors with concurrent significant bleeding risk, i.e. uncontrolled severe hypertension, bleeding diathesis, recent non-trivial spontaneous bleeding) (high risk of bleeding). | 2.2 (19/852) |
| C3: Aspirin plus clopidogrel as secondary stroke prevention, unless the patient has a coronary stent(s) inserted in the previous 12 months or concurrent acute coronary syndrome or has a high grade symptomatic carotid arterial stenosis (no evidence of added benefit over clopidogrel monotherapy). | 10.0 (85/852) |
| C4: Aspirin in combination with vitamin K antagonist, direct thrombin inhibitor or factor Xa inhibitors in patients with chronic atrial fibrillation (no added benefit from aspirin). | 1.9 (16/852) |
| C5: Antiplatelet agents with vitamin K antagonist, direct thrombin inhibitor or factor Xa inhibitors in patients with stable coronary, cerebrovascular or peripheral arterial disease (No added benefit from dual therapy). | 4.6 (39/852) |
| C6: Ticlopidine in any circumstances (clopidogrel and prasugrel have similar efficacy, stronger evidence and fewer side-effects). | 0 |
| C7: NSAID and vitamin K antagonist, direct thrombin inhibitor or factor Xa inhibitors in combination (risk of major gastrointestinal bleeding). | 2.6 (22/852) |
| C8: NSAID with concurrent antiplatelet agent(s) without PPI prophylaxis (increased risk of peptic ulcer disease). | 21.7 (185/852) |
| **Section D: Central Nervous System and Psychotropic Drugs** | |
| D1: Neuroleptics with moderate-marked antimuscarinic/anticholinergic effects (chlorpromazine, clozapine, flupenthixol, fluphenzine, pipothiazine, promazine, zuclopenthixol) with a history of prostatism or previous urinary retention (high risk of urinary retention). | 0 |
| D2: Selective serotonin re-uptake inhibitors (SSRI’s) with current or recent significant hyponatraemia i.e. serum Na+ < 130 mmol/l (risk of exacerbating or precipitating hyponatraemia). | 0.1 (1/852) |
| D3: Antipsychotics (i.e. other than quetiapine or clozapine) in those with parkinsonism or Lewy Body Disease (risk of severe extra-pyramidal symptoms). | 0.2 (2/852) |
| D4: Anticholinergics/antimuscarinics to treat extra-pyramidal side-effects of neuroleptic medications (risk of anticholinergic toxicity). | 0 |
| D5: Anticholinergics/antimuscarinics in patients with delirium or dementia (risk of exacerbation of cognitive impairment). | 0.4 (3/852) |
| D6: Neuroleptics as hypnotics, unless sleep disorder is due to psychosis or dementia (risk of confusion, hypotension, extra-pyramidal side effects, falls). | 0.1 (1/852) |
| D7: Levodopa or dopamine agonists for benign essential tremor (no evidence of efficacy). | 0.6 (5/852) |
| D8: First-generation antihistamines (safer, less toxic antihistamines now widely available). | 0.5 (4/852) |
| **Section E: Renal System. The following drugs are potentially inappropriate in older people with acute or chronic kidney disease with renal function below particular levels of eGFR (refer to summary of product characteristics datasheets and local formulary guidelines)** | |
| E1: Direct thrombin inhibitors (e.g. dabigatran) if eGFR < 30 ml/min/1.73m2 (risk of bleeding). | 0 |
| E2: Factor Xa inhibitors (e.g. rivaroxaban, apixaban) if eGFR < 15 ml/min/1.73m2 (risk of bleeding). | 0 |
| E3: NSAID’s if eGFR < 50 ml/min/1.73m2 (risk of deterioration in renal function). | 7.5 (64/852) |
| E4: Colchicine if eGFR < 10 ml/min/1.73m2 (risk of colchicine toxicity). | 0 |
| E5: Metformin if eGFR < 30 ml/min/1.73m2 (risk of lactic acidosis). | 0.2 (2/852) |
| **Section F: Gastrointestinal System** | |
| F1: Prochlorperazine or metoclopramide with Parkinsonism (risk of exacerbating Parkinsonian symptoms). | 0 |
| **Section G: Respiratory System** | |
| G1: Theophylline as monotherapy for COPD (safer, more effective alternative; risk of adverse effects due to narrow therapeutic index). | 0.2 (2/852) |
| G2: Non-selective beta-blocker (whether oral or topical for glaucoma) with a history of asthma requiring treatment (risk of increased bronchospasm). | 0.1 (1/852) |
| **Section H: Musculoskeletal System** | |
| H1: Non-steroidal anti-inflammatory drug (NSAID) other than COX-2 selective agents with history of peptic ulcer disease or gastrointestinal bleeding, unless with concurrent PPI or H2 antagonist (risk of peptic ulcer relapse). | 0.5 (4/852) |
| H2: Corticosteroids (other than periodic intra-articular injections for mono-articular pain) for osteoarthritis (risk of systemic corticosteroid side-effects). | 0.1 (1/852) |
| H3: NSAID with concurrent corticosteroids without PPI prophylaxis (increased risk of peptic ulcer disease). | 0.4 (3/852) |
| **Section J. Endocrine System** | |
| J1: Sulphonylureas with a long duration of action (e.g. glibenclamide, chlorpropamide, glimepiride) with type 2 diabetes mellitus (risk of prolonged hypoglycaemia). | 4.6 (39/852) |
| J2: Thiazolidenediones (e.g. rosiglitazone, pioglitazone) in patients with heart failure (risk of exacerbation of heart failure). | 0.1 (1/852) |
| J3: Oestrogens with a history of breast cancer or venous thromboembolism (increased risk of recurrence). | 0 |
| **Section K: Drugs that predictably increase the risk of falls in older people** | |
| K1: Benzodiazepines (sedative, may cause reduced sensorium, impair balance). | 8.2 (70/852) |
| K2: Neuroleptic drugs (may cause gait dyspraxia, Parkinsonism). | 2.2 (19/852) |
| K3: Hypnotic Z-drugs e.g. zopiclone, zolpidem, zaleplon (may cause protracted daytime sedation, ataxia). | 2.7 (23/852) |
| **Section N: Antimuscarinic/Anticholinergic Drug Burden** | |
| N1: Concomitant use of two or more drugs with antimuscarinic/anticholinergic properties (e.g. bladder antispasmodics, intestinal antispasmodics, tricyclic antidepressants, first generation antihistamines) (risk of increased antimuscarinic/anticholinergic toxicity). | 10.6 (90/852) |
